# Evaluating the Integration of Professional Nurse and Midwifery Advocacy Roles Within a Large Healthcare Organization: Assessing Service Utilization and Benefits—A Cross-Sectional Survey

**DOI:** 10.1155/jonm/5523523

**Published:** 2025-10-22

**Authors:** Naim Abdulmohdi, Andrea Tuckwell, Sally Goldspink, Hilary Engward, Nieky van Veggel, Marie Alexander

**Affiliations:** ^1^Faculty of Health, Medicine and Social Care, Anglia Ruskin University, Cambridge CB1 1PT, UK; ^2^East Suffolk and North Essex NHS Foundation Trust, Trust Offices, Colchester Hospital, Turner Road, Colchester CO4 5JL, Essex, UK; ^3^Faculty of Health, Medicine and Social Care, Anglia Ruskin University, Rivermead Campus, Chelmsford CM1 1SQ, UK

## Abstract

**Aims:**

To assess nursing and midwifery staff's awareness, use, and perceived benefits of the professional advocate (PA) role in clinical practice.

**Background:**

Nursing and midwifery staff face growing challenges, including heavy workloads, burnout, and staff shortages. In response to rising attrition and dissatisfaction, the professional nurse advocate and professional midwifery advocate roles were introduced to provide professional support, improve working conditions, and enhance staff well-being. However, there is limited evidence on their effectiveness, particularly regarding staff retention and well-being.

**Study Design:**

This evaluation employed a mixed-methods approach across four phases. This paper focuses on Phase 2, which involved a cross-sectional, self-report survey of 407 nursing and midwifery staff. Participants completed a self-administered questionnaire between October 2023 and January 2024.

**Findings:**

The study highlights significant gaps in awareness and access to PA services, with 64.9% of participants unaware of the role and 80.6% reporting no prior engagement. However, 95.3% expressed interest in future use, indicating strong positive attitudes toward the role and its support. Midwives reported greater familiarity with and access to PA services, likely due to longer exposure to the role, while nurses showed higher interest in repeated use. Informal communication channels, such as word-of-mouth, were prevalent, highlighting the need for more formal communication strategies. Participants valued the PA role for its emotional support, advocacy for staff rights, and career development, with confidentiality and accessibility identified as key priorities. Barriers to access included time constraints, concerns about confidentiality, and the need for greater organizational support. The PA role was perceived as empowering, positively influencing staff well-being and job satisfaction.

**Conclusions:**

Effective integration of PA services has the potential to empower nursing and midwifery staff, improving well-being and retention. Addressing access barriers and enhancing service delivery are essential to maximizing the impact of PA services in supporting staff well-being and professional growth.

**Summary**

• The study identifies critical gaps in PA service awareness and uptake, particularly regarding confidentiality concerns.
• Strengthening formal communication, ensuring secure and accessible services, and fostering managerial support are key to optimizing service use.
• The perceived benefits of PA services for staff well-being and professional development highlight their potential to create a more empowered and sustainable healthcare workforce.
• Investing in these services could enhance staff retention, morale, and the quality of care.

## 1. Introduction

The COVID-19 pandemic exacerbated the already high pressures on healthcare staff across various disciplines and settings, leading to unprecedented demands and excessive workloads. These challenges resulted in significant stress, with many healthcare workers experiencing anxiety, depression, and symptoms of posttraumatic stress disorder [1–3]. This situation highlights the urgent need for structured support to help healthcare staff's well-being, build resilience, and sustain effective patient care. Prolonged exposure to high-stress environments contributed to burnout, fatigue, and emotional exhaustion, impacting staff engagement [4], and increasing the likelihood of errors [5, 6]. Escalating levels of burnout among healthcare professionals have exacerbated staffing shortages, driven premature departures from clinical roles, and intensified challenges in staff retention [7, 8]; Royal College of Nursing [9]. Likewise, the RCN Foundation reported that nurses and midwives are at high risk of mental health issues, including depression and stress, due to their working conditions and the associated inability to deliver the expected level of care, often leading to feelings of self-blame for undesirable patient outcomes [6]. Similarly, West et al. [10] emphasized that the health and well-being of nurses and midwives are essential to the quality of care they provide. These factors, along with the substantial increase in nursing workload, pose serious risks to patient safety and are associated with higher patient mortality [11–13].

Prior to the COVID-19 pandemic, the UK National Health Service (NHS) recognized the need for stronger support systems to sustain a positive work environment. Initiatives such as the NHS Long Term Workforce Plan [14], the NHS Health and Well-being Framework [15], and Our NHS People Promise [16] demonstrated a commitment to staff well-being. However, the pandemic exposed persistent gaps, highlighting the need for targeted interventions to support staff mental health, retention, and resilience. In response, NHS England introduced the professional nurse advocate (PNA) role, based on the professional midwifery advocate (PMA) role, to support nursing staff. This aligns with workforce strategies, including the NHS Workforce Plan [14], to enhance retention through positive workplace cultures and workforce recognition. Globally, the WHO's Mental Health Action Plan (2013–2030) and Sustainable Development Goal 3 emphasize the need for resilient healthcare environments that protect staff well-being [59]. Nurses, representing over half the global healthcare workforce, were among the most affected during the pandemic, significantly influencing service delivery, patient outcomes, and care quality [18, 19]. The United Kingdom continues to face significant healthcare workforce challenges in nursing and midwifery, including persistent staff shortages, rising burnout, and high attrition rates among early-career professionals [20]. Recruitment has declined sharply, with a 34% drop in degree applications since 2021 [20, 21]. Retention remains a critical issue, with a 48.6% rise in NMC registrants leaving within 5 years [20]. Midwifery is experiencing significant workforce challenges, with 57% of surveyed midwives considering leaving the profession, according to a large survey [22, 23]. Midwifery faces a paradox of national shortages alongside limited employment opportunities for new graduates, driven by recruitment freezes. While the NHS Long Term Workforce Plan emphasizes domestic recruitment and retention, these strategies will take time to deliver impact, leaving the current workforce under considerable strain.

The professional advocate (PA) role is a key initiative in addressing some of these challenges, e.g., workforce burnout and fostering a positive, supportive work environment. However, limited research has explored its integration, utilization, and impact since its introduction during the COVID-19 pandemic.

## 2. Background

PA, as conceptualized in this context, involves defending, supporting, and promoting the interests and well-being of nurses and midwives. The roles of PNAs and PMAs, collectively referred to here as PAs, are central to fostering a supportive working environment. The PA role originated in midwifery following the UK statutory changes to supervisory practices [24] and was later extended to nursing [25]. It is underpinned by the advocating and educating for quality improvement (A-EQUIP) model, which is based on the works of Proctor [26] and Douglas and Ginty [27]. The A-EQUIP model was designed to empower nurses and midwives through advocacy, restorative clinical supervision (RCS), and professional development [28], with the addition of quality improvement [29]. Clinical supervision is well developed among the healthcare professionals and includes professional support and reflective learning with recognized benefits on the workforce [30, 31]. A detailed discussion of the model and advocacy can be found in previous papers [32, 33]. Through RCS, PAs help staff reflect on challenges and develop solutions, aiming to reduce stress and burnout. The normative function of the RCS promotes autonomy by enabling staff to assess and improve care practices, enhancing patient outcomes contributing to support staff structural empowerment. Formative supervision supports learning and development, helping nurses prepare for appraisals, revalidation, and role progression [34, 35]. PAs also drive quality improvement by encouraging proactive actions to deliver consistent, high-quality care. This model aligns with findings from The King's Fund [36], which highlights the benefits of autonomy in boosting job satisfaction, engagement, and well-being while reducing absenteeism and burnout.

Burnout, distinct from well-being, has significant implications for patient safety [37]. It manifests as prolonged work-related exhaustion, diminished motivation, and reduced personal achievement, often resulting in disengagement [38]. Empowerment can be examined through two primary lenses: structural and psychological [39], and this has been widely used in nursing research [40]. Structural empowerment (organization level) involves organizational strategies that provide staff with access to resources, support, and decision-making opportunities, fostering a supportive environment. It refers to the social structure at work that allows staff to achieve the work goals through accessing relevant information, support, resources, and opportunities [41]. It enhances psychological empowerment (individual level), which reflects an individual's motivation, autonomy, and belief in their ability to impact and influence outcomes. Empowered staff are more competent, autonomous, and purposeful, contributing to improved job satisfaction, reduced burnout, and better patient outcomes [39–43].

The PA role supports staff through RCS, enabling emotional processing, promoting psychological safety, and restoring thinking capacity; [34, 44]. In addition, supervision conversations help overcome barriers, enabling nurses to adapt to complex situations and improve their effectiveness [12]. Martin et al. [45] found that effective clinical supervision improves retention, enhances well-being and job satisfaction, and contributes to a resilient workforce. These findings on job satisfaction were consistent with those of Ash and Pollock [46], who reported similar outcomes among midwives using clinical supervision sessions with a PMA. However, McDonough et al. [47] cautioned against mandatory implementation without sufficient training and infrastructure, as the positive impact on ward culture and staff psychological safety can be inconsistent. According to the Royal College of Nursing [9], common reasons for nurses leaving the profession include feeling undervalued, excessive workload pressures, high stress, and inadequate management support.

Integrating the PA role into practice serves as a form of structural empowerment to enhance staff well-being, foster professional development, and recognize the value of staff contributions. Research indicates that increased organizational support is a critical factor in fostering personal resilience, while higher levels of personal resilience are a significant mediating factor in reducing the risk of staff burnout. Thus, greater organizational support and commitment are directly associated with enhanced resilience and a reduced likelihood of burnout [4, 48]. Moreover, improving working conditions not only boosts staff confidence in voicing concerns but also empowers them to influence service delivery and care quality [40]. Similarly, West et al. [10] identified key needs for nurses and midwives to support well-being and mental health, which are strongly linked to structural empowerment. These factors have been shown to improve staff psychosocial well-being and reduce turnover rates [39, 49], as well as affect quality of care and patient outcomes [12, 13].

Lees-Deutsch et al. [50] found that nurses supported by PAs reported positive effects on structural and psychological empowerment, with most survey questions receiving a median rating of 5 out of 6. Areas for improvement in restorative RCS included the need for time, a safe environment, and clearer communication about the process. Smythe et al. [51] identified work-related emotional distress (26.4%), professional relationship challenges (18.6%), and health and well-being concerns (15.7%) as the most common issues raised through the PA service. Supervisees valued the emotionally safe space, recognition of well-being concerns due to workload, and support for reflection and self-esteem development. Wade [52] reported similar perceptions among critical care nurses, emphasizing the PA role's importance in addressing workplace challenges through emotional and professional support. Similarly, Sharman et al. [53] highlighted the positive impact of the PA role on staff well-being, noting strong engagement with 508 PA support sessions over 21 months. The most frequent issues addressed included stress and well-being support, followed by staffing concerns and bullying. Similarly, Ash and Pollock [46] found increased and sustained engagement among midwives with clinical supervision sessions involving a PMA over time. Several discussion papers describe the PMA role and its perceived benefits; however, there is still limited primary research on its clinical integration or on evidencing these benefits [46, 54, 55].

## 3. Theoretical Framework

Based on service utilization models [56, 57], if staff perceptions of a service (e.g., the PA role) align closely with their needs, they are more likely to develop an interest in and positive attitudes toward accessing and utilizing the service. Awareness of a service (e.g., the PA service), along with its perceived ease of use and clear benefits, enhances its integration and effectiveness within clinical practice. Conversely, if the service is perceived as difficult to access, if there is limited knowledge about the PA role, or if it is seen as offering little benefit, it is unlikely to be adopted or effectively utilized.

**Figure 1:**
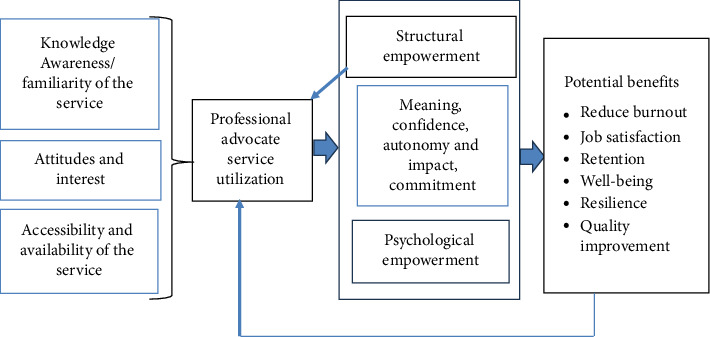
Study's theoretical framework.

A range of factors, including staff knowledge, awareness, attitude, and interest, shape how services are accessed and utilized [57]. Increasing awareness of the PA role's benefits among staff and organizations fosters acceptance, supports resource negotiation, and facilitates its integration into practice. As outlined above, the PA role has the potential to enhance both structural and psychological empowerment, leading to various benefits. However, further research is needed to provide evidence of this, evaluate its effects on nursing and midwifery practice, and explore its potential to drive improvements in care quality.  Figure 1 illustrates the interconnections between the key concepts of this study, highlighting the factors influencing service utilization, potential benefits, and possible links to the empowerment model described above [39, 41].

## 4. Methods

### 4.1. Aim and Research Design

The purpose of this project was to explore and understand the impact (if any) of the PNA/PMA role on nurse and midwife quality improvement in practice, well-being, and the retention of nurses and midwives within a large healthcare organization part of the NHS. To achieve this, a four-phase mixed-methods study was conducted, and the research presented here forms part of a larger project [32]. The objective of this phase was to examine how nurses and midwives in a large healthcare organization access and utilize the PA service, as well as their perceptions of the PA role's benefits for their well-being and retention. This phase used a self-report and cross-sectional survey design including quantitative and qualitative data collection. It is reported in accordance with the Consensus-Based Checklist for Reporting of Survey Studies (CROSS) [53].

### 4.2. Sampling and Data Collection

Registered nurses and midwifery staff employed at a large healthcare organization in England were invited to participate through multiple communication channels, including the organization's newsletter, internal communication boards, printed invitation posters distributed across the organization, and emails sent by the communication team. In addition, a clinical research coordinator visited various wards with digital devices to facilitate survey completion for interested staff [58]. Data were gathered using an anonymized self-report questionnaire administered through a secure online platform provided by the Joint Information Systems Committee (JISC) (https://www.onlinesurveys.ac.uk/). The online survey comprised three initial sections: a participant information sheet (PIS), a consent section, and the researcher's contact details. Following the initial invitation, three subsequent email reminders were sent to prompt staff to complete the survey. The survey was conducted between 1 October 2023 and 15 January 2024.

Study participants were recruited using a purposive sampling technique. The target population included registered nurses and midwives working within a large UK NHS healthcare cluster encompassing primary, secondary, and tertiary care settings. The sampling frame consisted of 4000 eligible nursing and midwifery staff. For this study, the sample size was determined to be 385, considering a 50% response rate, a 95% confidence interval, a significance level of 0.05, and a margin of error of 5% [59]. To account for potential nonrespondents and missing data, a total of 407 registered nurses and midwives were recruited. The survey was pilot tested on a sample of 10 participants to ensure the face validity of the tools and the clarity of the survey content. These participants provided feedback on the clarity, readability, and relevance of the questions, including how they interpreted and answered each item. Based on their input, slight refinement was made to four questions to improve clarity and consistency.

### 4.3. Survey

The survey content was informed by discussions with stakeholders from the local healthcare organization and a literature review assessing the utilization of PA services and their perceived impact on staff well-being. The survey design was guided by the A-EQUIP PA model [14] and theoretical frameworks such as structural empowerment [39] and Andersen's [57] service utilization model. An experienced nurse researcher initially developed the survey items based on the literature and discussions with clinical collaborators from the nursing and midwifery team [32, 33]. These were subsequently reviewed by the team, with some items removed, edited, or newly added by a panel of experts, including an academic specializing in PA, two senior researchers, senior clinical staff with experience in PA, and a nurse leader. This approach was used to establish the content validity of the survey. The following section describes the content of the survey. The questionnaire comprised four sections, as follows.

#### 4.3.1. Demographic Characteristics

The first part gathered demographic information, including age, employment type, clinical setting (e.g., clinical or nonclinical), specialty, length of service, and professional rank.

#### 4.3.2. Awareness, Access, and Service Utilization

The second part of the survey comprised 20 questions focusing on staff awareness, access, and utilization of PA services based on the theoretical framework described in Figure 1. This included the frequency and method of access to PA services, staff attitudes toward the service, and their intention to use it again (as detailed in Appendix Table A1). Participants' awareness of the role was assessed using six questions: three multiple-choice questions, two utilizing a 6-point Likert scale to gauge their level of agreement regarding their awareness of the role, and one open-ended question. Access to and utilization of the service were evaluated through eight questions: four multiple-choice questions, one employing a 6-point Likert-type item, ranging from 1 (*strongly disagree*) to 6 (*strongly agree*) to measure agreement on the ease of access to the service, one question using a four-point Likert-type item, ranging from 1 (*never*) to 4 (*often*) to assess the frequency of access, and finally, two open-ended questions. Attitudes toward accessing and using the service were measured using five questions: two multiple-choice questions and three employing a 6-point Likert-type, ranging from 1 (*strongly disagree*) to 6 (*strongly agree*) to assess participants' attitudes toward using the service. The Likert-type questions provided scores for each section, including awareness, ease of access, frequency of access, and attitudes. The survey included open-ended questions about barriers to accessing the service, preferred methods of access, and suggestions for improving its relevance and effectiveness.

#### 4.3.3. Perceived Benefits of PA

The third part included questions about the perceived benefit of the PA role on various aspects, such as well-being, burnout, satisfaction, and retention. It also assessed the component of psychological empowerment based on Laschinger et al. [39] Lees-Deutsch et al. [56], four items based on the A-EQUIP model domains [25] and other factors such as burnout [60] employing a 6-point Likert-type item, ranging from 1 (*strongly disagree*) to 6 (*strongly agree*) (Table 1).

### 4.4. Ethical Considerations

The study received ethical approval from Anglia Ruskin University Faculty Research Ethics Panel, with the approval number: ETH2223-9518. The research was conducted in accordance with the Declaration of Helsinki (1964). Participation in this study was voluntary. The first page of the online survey introduced the study, followed by a page containing the PIS and the researcher's contact details. The third page included a consent statement, which each participant confirmed digitally, indicating that they had read the PIS and consented to participate in the study. This constituted digital informed consent, rather than handwritten consent, which is a common procedure [61]. These procedures were approved by the university ethics panel.

### 4.5. Data Analysis

Data analysis was performed using Version 28 of the Statistical Package for Social Sciences (SPSS). The data were quantified through means, medians, frequencies, and standard deviations (SDs). Initially, histograms and Shapiro–Wilk's test were employed to assess the approximate normality necessary for parametric tests. As the datasets did not meet the assumptions for parametric analysis, nonparametric statistical tests were carried out. Spearman's correlation coefficients were utilized to assess the correlations between demographic variables and key study variables (see Results section). To investigate any difference and the impact of demographical factors on staff awareness, level of access, and potential impact of PA, the Kruskal–Wallis test was conducted. The statistical significance was set at an alpha level of *p* < 0.05.

The content of the open-ended questions in the survey was analyzed using descriptive thematic analysis, following the six phases outlined by Braun and Clarke [62]. Initially, all responses were read in one sitting to develop familiarity with the data, and preliminary notes were made. The transcripts were then exported from the online survey platform and imported into NVivo 14 software to facilitate systematic coding. Coding was conducted inductively and iteratively, with all data reviewed multiple times to enhance the validity of the coding. All transcripts were coded in a systematic way, generating a list of descriptive codes. The codes were then clustered together to identify meaningful patterns. These categories were reviewed and refined into initial subthemes, which were subsequently developed into overarching themes. The analysis focused on exploring staff awareness and access to the service and on identifying suggestions for improving relevance, in order to add contextual depth to the quantitative findings. Each anonymized response was assigned a participant number, ranging from 1 to 407, to facilitate the referencing of quotations. For example, the 10th response was denoted as participant ten (P10).

## 5. Results

The following section initially presents the quantitative results of the survey, followed by a section on qualitative findings of the survey.

### 5.1. Quantitative Results

#### 5.1.1. Descriptives

Table 2 illustrates that 66.1% (*N* = 269) of respondents are primarily nurses, predominantly in the adult field (51.4%; *N* = 209), while 30.5% (*N* = 124) are midwives, and 3.4% (*N* = 14) hold dual roles as both nurses and midwives. The majority (85.5%; *N* = 348) of respondents work in clinical settings, with over half (57.2%; *N* = 233) employed on a full-time basis. Most participants hold positions either as staff nurses (34.2%; *N* = 139, NHS Band 5) or midwives or senior nurses (39.6%; *N* = 142, Band 6). Notably, 95.6% (*N* = 389) of respondents do not currently have a PA role. The average age of participants is 39.94 years, with an average of 14.13 years of professional experience. By December 2022, within this large healthcare organization, there were 13 PNAs and 7 PMAs in post. This increased to 20 PNAs and 12 PMAs by September 2023. With approximately 3730 nurses and 270 midwives employed in this healthcare organization, this equated to a ratio of 1 PNA to 287 nurses and 1 PMA to 39 midwives in December 2022, improving to 1:187 (PNA) and 1:23 (PMA) by September 2023.

#### 5.1.2. Knowledge, Attitudes, and Access

Nearly two-thirds of the participants (64.9%; *N* = 264) were not aware of the exact role of the PA, and only 55.8% (*N* = 227) were familiar with PA services in general, indicating a limited current knowledge and awareness of these services (Table 3). Most of the participants (80.6%; *N* = 328) did not access the PA services, while among those who did (17.9%; *N* = 73), the majority (99%; *N* = 72) expressed strong interest in accessing the service again. In addition, nearly all participants (95.3%; *N* = 388) indicated an interest in accessing PA support if it became available to them. Nurses and midwives moderately agreed that they would recommend the service to colleagues and strongly disagreed that the service lacks benefits. There was a slight agreement that it is easy to access, and leadership encourages the use of the service. Most of the participants had no preference on whether to meet a PA who is known or unknown to them (58%; *N* = 236), followed by a preference of a third of the sample for seeing someone they know (30.2%; *N* = 123). Most of the participants who accessed the service used self-referral rather than colleague or manager referral. Overall, despite limited access and support from direct managers, participants showed strong positive attitudes toward the PA service.

The level of awareness was higher among midwives with a statistically significant difference in the level of awareness about the services between nurses and midwives (*H* [2] = 94.1, *p* < 0.001). Midwives exhibit greater familiarity (*H* [2] = 45.89, *p* < 0.001), with a higher level of access (*H* [2] = 27, *p* < 0.001), while nurses demonstrate more positive attitudes toward repeated access (*H* [2] = 36.8, *p* < 0.001). This could be explained by the availability of PA to midwives for the last 7 years compared to 2 years for nurses at the time of conducting this research. Both nurses and midwives expressed similar interest in accessing the service (*H* [2] = 0.972, *p*=0.615).

**Figure 2:**
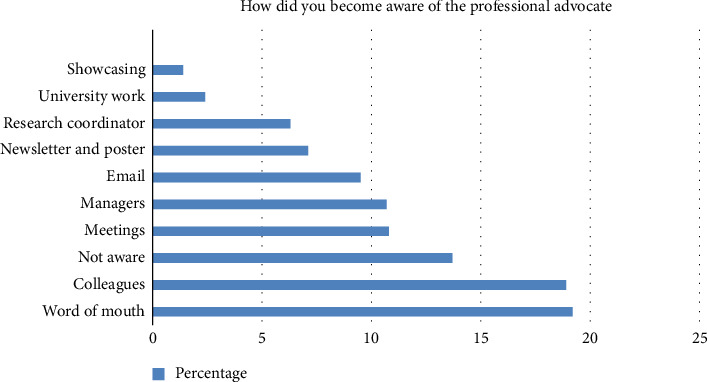
Methods by which staff became aware of the service.

**Figure 3:**
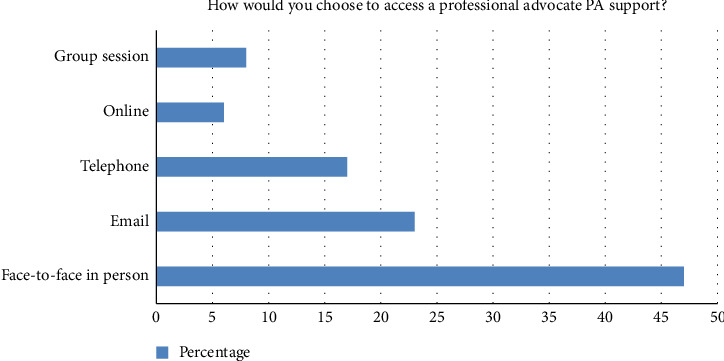
Methods of staff preference for accessing the service.

The multiple-choice questions demonstrated that the most frequent methods of raising awareness about PA were word of mouth (19.2%) and colleagues (18.9%) (Figure 2). This suggests that informal networks play a key role in disseminating information about the role of PAs more than formal communication, and perhaps there is more work required to increase staff awareness of the service via formal communication channels. There are several potential reasons behind this trend. Most participants (47%) preferred face-to-face sessions, likely due to the nature of the issues being addressed (Figure 3). The emotional and complex nature of advocacy makes individuals more comfortable discussing sensitive topics directly with a PA, fostering greater trust and understanding.

#### 5.1.3. Perceived Benefit and Usefulness of the Service

Table 4 shows Cronbach's alpha, the mean score, and SDs for all the scales used in the survey. The results illustrate an excellent level of reliability, demonstrated by Cronbach's alpha being equal to or above 0.90 for all the items used. The overall mean score of perceived benefit was 77.36 (SD = 15.93) on a scale of 16–96, indicating a high level of overall perceived benefits of PA on the scale domains. The mean score of the impact of using the A-EQUIP model (enhancing the leadership on service improvement, contributing to patient care and safety, ability to improve practice, enhancing professional development) was 19.16 (SD = 3.93) on a scale of 1–24, which again indicates a high level of perceived benefit. Participants perceived that PA would have a high positive influence on empowerment (mean = 33.66, SD = 7.72), reducing burnout (mean = 9.46, SD = 7.72), increasing satisfaction (mean = 4.89, SD = 1.11), retention (mean = 5.02, SD = 1.09), and psychological well-being (mean = 5.17, SD = 0.99). Overall, participants perceived the PA service to be clinically useful and believed it could positively impact their well-being, retention, and ability to enhance patient care.

Although both nursing and midwifery participants reported high potential benefit as discussed above, nurses perceived the service to have a higher level of benefit compared to midwives, which was statistically significant. The findings reveal significant differences between nurses and midwives across several key areas. In terms of overall benefit, nurses reported that the service had a greater effect in empowering their influenceover their work compared to midwives, with a statistically significant result (*H* [2] = 16.25, *p* < 0.001). This pattern was similar for the meaning of work, where nurses reported that the service contributed to a stronger sense of meaning compared to midwives (*H* [2] = 18.55, *p* < 0.001). Regarding staff impact and influence, nurses' believe the PA will have more influence on their roles than midwives (*H* [2] = 12.19, *p* < 0.002). Nurses reported greater benefits of the service in relation to burnout compared to midwives (*H* [2] = 20.1, *p* < 0.001). A significant variation was also observed in the A-EQUIP domains, with nurses higher perceived benefit in those domains compared to midwives (*H* [2] = 7.18, *p* = 0.028). Similarly, nurses reported greater satisfaction in their roles compared to midwives (*H* [2] = 9.93, *p* = 0.007), as well as more confidence (*H* [2] = 14.41, *p* < 0.001) and autonomy in their work (*H* [2] = 9.48, *p* = 0.009).

No significant difference was found in terms of well-being across the groups (*H* [2] = 1.43, *p*=0.490). However, nurses showed a stronger intention to stay in their roles compared to midwives, with a significant difference (*H* [2] = 7.68, *p*=0.022). These results suggest that nurses consistently report higher levels across domains such as overall perceived benefit, burnout, satisfaction, and retention compared to midwives.

### 5.2. Qualitative Analysis

**Figure 4:**
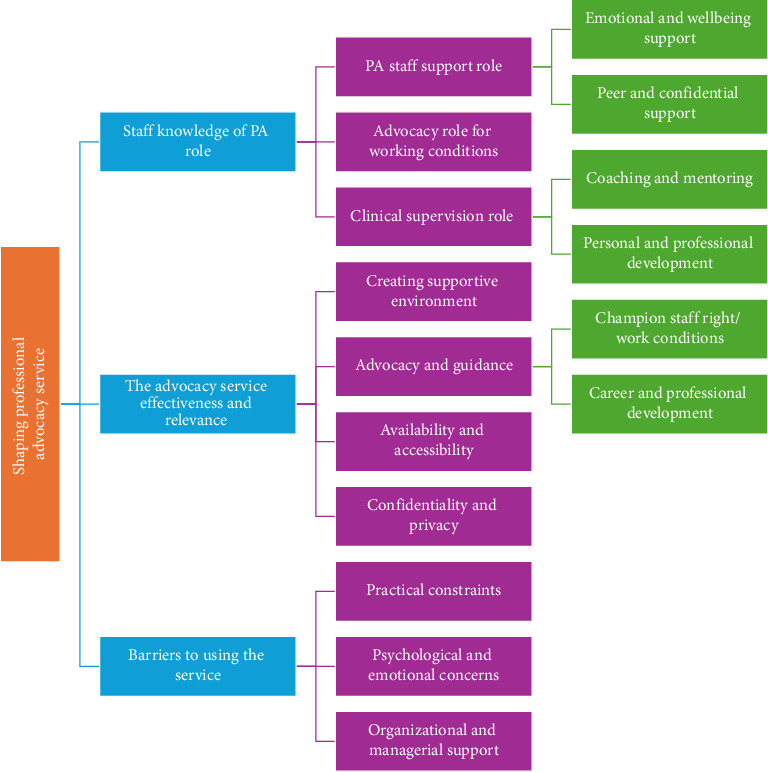
Themes and subthemes on staff perceptions and experiences of the professional advocacy service.

The analysis of the participants' responses to the open-ended question results in three major themes regarding staff perceptions and experiences of the PA service. These themes illustrate how the PNA and PMA services could be used to shape and enhance service integration, effectiveness, and utilization (Figure 4).

#### 5.2.1. Staff Knowledge of the PA Service

Most participants described the PA role as primarily providing essential support to nursing and midwifery staff, with emphasis on emotional, confidential, and peer support. Emotional support was identified as the advocate's main responsibility, aiding staff in managing stress, burnout, and compassion fatigue. Advocates foster a safe environment for open discussions, facilitate debriefing after critical incidents, and promote self-care and resilience strategies. Confidential support was equally valued, with advocates offering a nonjudgmental space for addressing interpersonal conflicts and personal issues.

*“A PMA provides support both professionally and emotionally; it is a supportive, nonjudgmental role.” (P187)*

*“To support workplace, debrief after an incident and to ask for help when feeling psychologically unsafe to speak to managers and for personal development conversations.” (P213)*

Beyond their supportive functions, advocates were seen to play a key role in clinical supervision and advocating for improved resources and working conditions. Regular supervision sessions facilitated staff reflection on practice, ethical dilemmas, and patient safety concerns.

*“To provide a safe space for a conversation about work-related or personal matters.” (P76)*

*“To provide a safe no-judgmental space where staff can explore clinical experiences and reflect.” (P402)*

However, participants made limited connections between the advocate's role and direct improvements in patient care. While their contributions to clinical supervision and professional development were acknowledged, the advocate role was primarily viewed as supporting staff well-being rather than driving quality improvement in clinical practice.

#### 5.2.2. Making the Advocacy Service Effective and Relevant

Participants' responses highlight the usefulness of the PA service in a few key domains. Five subthemes emerged as central to staff expectations: supportive environment and well-being, advocacy and guidance, availability and accessibility, confidentiality and privacy, and career and professional development. Most participants emphasized the importance of fostering a supportive environment and enhancing staff well-being. Advocates are expected to provide emotional support during challenging times, helping staff manage workload pressures, burnout, and compassion fatigue. Staff also expect advocates to promote resilience through well-being sessions, mental health support, and welfare initiatives.

*“A supportive service 7 days a week where I can contact for me or signpost another colleague to for help.” (P137)*

Advocacy and guidance were also highlighted as critical. Advocates are expected to champion staff rights, address workforce challenges, and offer support in ethical decision-making.

*“Speaking up for me and helping me to speak up.” (P72)*

Some participants also valued the role of advocates in facilitating career and professional development by identifying opportunities, ensuring fair access to training, and supporting growth.

“Fair access to training opportunities.” *(P212)*

The availability and accessibility of the service were seen as essential. Staff expect advocates to be consistently present and visible in the workplace, fostering trust and encouraging engagement.

“Regular visible known team in the work environment who I can contact when I am struggling.” *(P101)*

Confidentiality and privacy were equally vital. Participants expect advocates to offer impartial advice while safeguarding sensitive information, creating a safe and trusted environment.

“Assured confidentiality when seeking support.” *(P186)*

#### 5.2.3. Barriers to Accessing the Service

Barriers to accessing PA services were grouped into three overarching subthemes: practical constraints, psychological and emotional concerns, and organizational and managerial support. These subthemes highlight the multifaceted challenges staff face when seeking advocacy support. Practical constraints emerged as significant barriers, with time and logistical challenges frequently cited. Participants noted that demanding workloads and inflexible schedules often prevented them from accessing services. Geographical distance further compounded these issues, making it difficult to attend sessions located far from their workplace or residence.

*“Lack of time away from clinical work.” (P302)*

Psychological and emotional concerns also presented substantial barriers. Many participants expressed fears about confidentiality, worrying that information shared might be leaked to managers or colleagues, potentially harming their reputation or career.

*“Worried about my manager finding out.” (P43).*

Embarrassment and discomfort about seeking help also featured prominently, as participants feared being perceived as weak. Addressing these deeply personal concerns is crucial for fostering a safe and nonjudgmental environment. Hesitation to seek support was further influenced by fears of judgment or being labeled a troublemaker.

*“Scared to speak up for fear of reprisal and repercussions.” (P172)*

Organizational and managerial support was the third major theme. A lack of encouragement from managers and limited service availability during working hours were significant barriers. Managerial indifference further exacerbated access issues.

*“Lack of availability… If not allowed time out in working day to seek advice.” (P99)*

*“My line manager didn't actively support me.” (P223)*

These findings highlight the critical role of organizational culture and leadership in enabling access to advocacy services, emphasizing the need for visible, active support from management to normalize and encourage engagement.

## 6. Discussion

The findings from our survey provide a detailed understanding of nursing and midwifery staff access, perceptions, knowledge, and the perceived usefulness of PA services within a large healthcare organization. The study highlights that participants view the role of the PA as enhancing their well-being and professional development, while also identifying gaps in awareness and access that could limit the service's effectiveness.

### 6.1. Utilization of PA Services and Associated Barriers

This study shows that PMA provision is approaching the target ratio of one advocate per 20 staff (1:20), whereas PNA coverage, though continuing to expand, remains well below this level. Only 4.4% of participants currently hold PA roles, and 80.6% have never accessed the service, with just 17.9% reporting any usage. These low utilization rates may reflect limited awareness of the role, as only 35.1% of respondents were aware of the service's availability and 55.8% were familiar with its offerings, as well as the early stage of its implementation in the surveyed organization. Qualitative findings in our study identified important barriers that can explain the low level of service utilization, with time constraints due to workload cited most frequently, along with a gap in staff knowledge and familiarity with the role, and concerns about being perceived as weak. This is consistent with findings by Ash and Pollock [46], who reported low engagement with clinical supervision among midwives during the first year of the PMA service, followed by a significant improvement in subsequent years. In contrast, Lees-Deutsch et al. [50] reported relatively higher access rates among UK nurses, with 57.5% receiving RCS from a PA. Despite low uptake of PA services in our study, interest in the services was strong, with 95.3% of participants expressing a desire to access the service if available, and 99% of previous users indicating a willingness to use it again. This aligns with findings by Sharman et al. [53], where 508 staff accessed the service over 21 months, and 16% returned for repeat support.

Other barriers identified in our qualitative findings to accessing PA services included concerns about confidentiality, fear of repercussions, service availability, and limited awareness of how to access the services. These findings echo those of previous studies, which identified practical and psychological barriers as key limitations to workplace support initiatives. For example, time constraints in attending PA support have been described as disempowering for staff [50]. Similarly, Sharman et al. [53] identified time constraints as a major barrier for both staff accessing the service and PAs delivering the service. These barriers are consistent with similar literature on clinical supervision, with time, workload, and space identified as the main obstacles to both engaging in and delivering effective clinical supervision [30, 31, 46]. In addition, reliance on informal communication channels such as “word of mouth” in our study highlights the need for structured awareness-raising strategies and leadership support. The Royal College of Nursing [9] has similarly identified managerial support, staff valuation, and manageable workloads as critical factors influencing retention. These barriers could potentially affect the effectiveness of the PA service and its role in fostering a supportive culture, empowering staff and raising awareness of quality improvement.

A notable difference emerged between nurses and midwives: midwives reported greater familiarity with and access to PA services, while nurses expressed more positive attitudes toward reaccessing the service. This could suggest that while midwives benefit from longer-term access, as it has been integrated into practice for the past 8 years, nurses may perceive the service as more beneficial or be more optimistic about its potential, possibly due to their relatively newer exposure to it in the past 2 years. Since 2017, PMAs have reported monthly data to the Department of Health. Referral and reporting processes for both roles in the local organization were integrated in 2023, allowing leads PA to triage requests for advocacy support across nursing and midwifery. Although the PMA service was, from its inception, linked to maternity governance structures and used to escalate concerns around patient safety, workload, and staffing, there was no formal strategy guiding either service. A referral and escalation strategy is now being introduced to the local healthcare organization.

### 6.2. Perceived Benefits and Usefulness of PA Services

The overall mean score for the perceived benefits of PA services was high, with respondents associating the role with improvements in staff empowerment, burnout reduction, job satisfaction, retention, and psychological well-being. These findings are also consistent with the study's qualitative data on staff perceptions of the PA role and their expectations of PA services, highlighting their usefulness and relevance. These results align with recent studies [50–53], which demonstrate high satisfaction levels and positive impacts on practice and well-being among supervisees. In addition, the broader literature supports the link between empowerment, organizational support, and clinical supervision in improving staff well-being, reducing burnout, and enhancing retention; Mansour and Mattukoyya, 2018; [4, 45]. West et al. [10], in their research, identified three key needs for nurses and midwives to support well-being, sustain motivation, and minimize workplace stress: autonomy, belonging, and contribution. Meeting these needs empowers staff and supports not only their well-being but also the delivery of safe and effective care. The PA role, through RCS sessions, facilitates reflective practice and fosters compassionate leadership, aiming to improve working conditions, reduce staff stress, and enhance patient safety through staff engagement and contribution to quality improvement. These outcomes are particularly important in high-pressure clinical settings, where staff stress and poor working conditions can compromise the delivery of safe care. Participants in our study primarily associated the PA service with staff well-being and professional development rather than direct contributions to patient care. Similarly, Sharman et al. [53] noted that only 18% of PA service utilization was related to quality improvement, with the majority focused on staff support and career development. Access to professional development opportunities and the ability of staff to influence their work environment have been identified as significant factors in enhancing staff retention [63]. Participants in our study focused their responses on the usefulness of the service for their well-being and professional development, with limited reference to how it contributes to patient care. This highlights a need to raise awareness of the broader benefits of PA services in improving the quality of care.

Moreover, nurses in our study reported a higher perceived benefit of the service compared to midwives, suggesting that while both groups recognize its advantages, nurses may feel a greater need for or derive more value from it. Our quantitative and qualitative findings highlight the importance of visible advocacy roles, managerial encouragement, and organizational support in fostering trust and increasing access to PA services. Participants expressed a preference for face-to-face interactions (47%), underscoring the significance of personal engagement in building trust. This aligns with Sharman et al. [53], where 65% of PA sessions were delivered in person. Limited awareness, familiarity, and availability of the services, coupled with key barriers such as time constraints, concerns about confidentiality, the service being in its early stages of development, and the need for managerial support, are significant factors affecting access to and utilization of the PA services as illustrated in our study. Overall, all staff expressed positive attitudes and perceived the PA support as having a benefit, making it a key driver for investment in the development of the service to better meet their needs. While attitudes toward the service are positive, structural and cultural barriers remain significant impediments to its full utilization. Ensuring that advocacy services are accessible, confidential, and actively supported by leadership is essential to addressing staff needs and effectively overcoming these barriers.

### 6.3. Strengths and Limitations

The survey design, incorporating both quantitative data and free-text qualitative responses, provided deeper insights into the perceptions, utilization, impact, and barriers associated with PA roles. The survey was co-designed with input from stakeholders, clinical staff, and experts, ensuring its relevance to practice. However, the study has several limitations. The reliance on self-reported data may introduce response bias, and the cross-sectional design limits causal inferences. In addition, as the study was conducted within a single organization, the findings may not be fully representative. Awareness of and access to PA services may vary across organizations, potentially affecting the generalizability of the results. Although this study used an anonymous survey designed to enable all staff to provide anonymized responses freely, it is important to acknowledge that the most disempowered individuals may still have been less likely to participate. Future research should consider more inclusive methods, such as interviews, peer-facilitated discussions, or participatory approaches, to better capture these perspectives. Furthermore, the study did not fully capture the perspectives of organizational leaders, whose support is critical for the effective implementation of PA roles. At the time of the survey, PA services were still in their early stages, having been implemented only 2 years prior. Future studies should address these limitations to enhance the robustness and applicability of the findings and consider experimental designs to evaluate the effectiveness of this service.

## 7. Conclusion

The survey highlights the perceived usefulness of the PA role in supporting nursing and midwifery staff, particularly in providing emotional support, advocacy, and professional development. Despite these perceived benefits, there is a significant gap in awareness and access to these services, with only a small percentage of staff currently utilizing them. Both nurses and midwives viewed the impact of these roles as positive, with nurses reporting a slightly higher perceived benefit. However, barriers such as time constraints, concerns about confidentiality, and limited awareness hinder broader access to these services. To maximize staff use and the benefits of PAs, managers and leaders must improve visibility, remove barriers, and strengthen formal communication to raise awareness. For instance, protected time could be built into staff schedules to allow access to PA without adding workload pressure. Reassurance around confidentiality may be supported through clear policies and anonymized staff testimonials. Visibility can be enhanced by embedding the service in staff induction, regular briefings, and peer support initiatives; normalizing the use of the service; and establishing communication platforms such as newsletters and intranet banners. Together, these actions could improve service utilization and strengthen support for nursing and midwifery safety staff.

## Figures and Tables

**Table 1:** Professional advocate survey items on perceived benefits.

For each item, please select the option below that best indicates how much you agree with the following statements. The PMA/PNA support will	
Item statement	Domain
Enhance my role in leading on service improvement	A-EQUIP
Enhance my contribution to patient care and safety	
Enhance my abilities to improve my practice	
Enhance my professional development	

Increase my job satisfaction	Job satisfaction

Have a positive influence on my retention in my organization	Retention

Have a positive influence on my well-being	Well-being

Reduce my feeling of exhaustion from work	Burnout
Reduce my feeling of disengagement with work	

Have increased my feeling of being valued by my organization (being valued)	Empowerment
Increase my feeling of the importance of my job to me (meaning of work)	
Increase my feeling that the work I do is meaningful to me (meaning of work)	
Increase my confidence in my abilities to do my job (confidence)	
Increase my feeling of autonomy in determining how I do my job (autonomy)	
Increase my impact on what happens in my department (impact and influence)	
Increase my feeling that I have significant influence over what happens in my department (impact and influence)	

**Table 2:** Demographic.

**Characteristics**		**Number (%)**	

Profession	Nurse	269 (66.1)	
	Midwife	124 (30.5)	
	Nurse and midwife	14 (3.4)	

NMC registration	Adult	209 (51.4)	
	Mental health	6 (1.5)	
	Pediatric	43 (10.6)	
	Learning disability	1 (0.2)	
	Midwife	124 (30.5)	
	Dual registration	24 (5.9)	

Work settings	Mainly clinical	348 (85.5)	
	Mainly nonclinical	27 (6.6)	
	Combination of clinical and nonclinical	30 (7.4)	
	Other	2 (0.5)	

Contract	Full time	233 (57.2)	
	Part time	162 (39.8)	
	Staff bank	12 (2.9)	

Job title	Staff nurse	139 (34.2)	
	Senior nurse or midwife	142 (34.9)	
	Sister or charge or senior midwife	44 (10.8)	
	Advanced practice or specialist	40 (9.8)	
	Manager	22 (5.4)	
	Research or education	12 (2.9)	
	Other	8 (2.0)	

Range of clinical experience (years)	0–5	115 (28.3)	
	6–10	71 (17.4)	
	11–15	74 (18.2)	
	16–20	48 (11.8)	
	21–25	34 (8.4)	
	26 and above	65 (16.0)	

Advocacy role	Not a PNA/PMA	389 (95.6)	
	PMA/PNA	18 (4.4)	

**Characteristics**	**Mean (SD)**	**Range**	
		**Minimum**	**Maximum**

Age (years)	39.94 (11.48)	20	70
Experience (years)	14.13 (11.80)	1	53

**Table 3:** Knowledge, attitude, and access.

**Characteristics**		**Number (%)**	

Awareness of a local PA	Not aware	264 (64.9)	
	Aware	143 (35.1)	

Familiarity with the role	Unfamiliar	180 (44.2)	
	Familiar	227 (55.8)	

Accessed PNA/PMA support	No	328 (80.6)	
	Yes	73 (17.9)	
	Other	6 (1.5)	

Initiate contact	Self-referral	59 (14.5)	
	A colleague recommended	13 (3.2)	
	Other	7 (1.7)	
	Did not access	328 (80.6)	

Preference of PA	Known	123 (30.2)	
	Unknown	48 (11.8)	
	No preference either	236 (58)	

Frequency of access in the last year	Never	334 (82.1)	
	Rarely	43 (10.6)	
	Sometimes	28 (6.9)	
	Often	2 (0.5)	

Interest in accessing the service	Interested	388 (95.3)	
	Not interested	11 (2.7)	
	Prefer not to say	8 (2)	

Would you seek their support again in the future?	No	2 (0.5)	
	Yes	77 (18.9)	
	Didn't access	328 (80.6)	

**Characteristics**	**Mean (SD)**	**Range**	
		**Minimum**	**Maximum**

Effort to access (how easy)	3.49 (1.89)	1	6
Access is encouraged by leadership	3.2 (1.96)	1	6
Recommend PNA/PMA to my colleagues	4.9 (1.4)	1	6
Can't see any benefits from it	1.68 (1.23)	1	6

**Table 4:** Perceived benefit of the PA role on variables.

Characteristics	Mean (SD)	Range		Cronbach's alpha
		Minimum	Maximum	
Overall potential benefit of the PA role across all variables	77.36 (15.93)	16	96	0.976
A-EQUIP	19.16 (3.93)	4	24	0.949
Structural_ empowerment	33.66 (7.72)	7	42	0.964
Meaning of work	9.78 (2.33)	2	12	
Staff impact and influence	9.73 (2.27)	2	12	
Being valued	5.00 (1.13)	1	6	
Confidence	4.96 (1.14)	1	6	
Autonomy	4.78 (1.21)	1	6	
Burnout	9.46 (2.52)	2	12	0.944
Exhaustion	4.67 (1.35)	1	6	
Disengagement	4.79 (1.25)	1	6	
Satisfaction	4.89 (1.11)	1	6	
Retention	5.02 (1.09)	1	6	
Well-being	5.17 (0.99)	1	6	

**Table 5:** Professional advocate survey items on awareness, access, and attitudes.

Aspect	Item	Method or response
Awareness	Are you familiar with the role of the professional advocate?	Yes or no
	Do you know of a named professional advocate in your department?	Yes or no
	You are aware of the role of the PNA/PMA in your department?	6-point Likert-type
	Your colleagues are aware of the PMA/PNA? Role in your department?	6-point Likert-type
	How did you become aware of the professional advocate (PNA/PMA) within your department/specialty? (Select all applicable)	Meetings, word of mouth showcasing events, newsletters, email, colleagues, managers, not applicable (not aware), other
	What is the role of PNA/PMA in your opinion?	Open-ended question

Access and utilization	Have you accessed the professional advocate (PNA/PMA) support?	Yes, no, prefer not to say
	It is easy to access PNA/PMA support	6-point Likert-type
	If you used the professional advocate (PMA/PNA) support, how did you initiate contact with them?	You directly contacted a PNA/PMA A colleague suggested it to you Not applicable (did not access this service) Other
	In what format did you receive the professional advocate (PMA/PNA) support? (Select all relevant)	
	How many times have you used the PNA/PMA service in the last year?	Often Sometimes Rarely Never
	How would you choose to access a professional advocate (PMA/PNA) support?	Open ended
	What were your reasons for seeking a PMA/PNA? (Select all relevant)	Emotional support Revalidation Career advice/conversation Professional support Safeguarding Critical incident/serious case review Civility conversation
	What would stop you from seeing a PMA/PNA?	Open ended

Attitudes to use the service	If the professional advocate (PMA/PNA) support made available to you, would you be interested in using this support when needed?	Yes, no, prefer not to say
	If you have already used PMA/PNA, would you seek their support again in future?	Yes, no, did not use PA, prefer not to say
	I would recommend the PMA/PNA to my colleagues.	6-point Likert-type
	Accessing PMA/PNA support is encouraged by my department	6-point Likert-type
	I cannot see any benefits in attending PNA/PMA support	6-point Likert-type

## Data Availability

Data supporting the findings of this study are available on request from the author.

## References

[1] Pappa S., Ntella V., Giannakas T., Giannakoulis V. G., Papoutsi E., Katsaounou P. (2020). Prevalence of Depression, Anxiety, and Insomnia Among Healthcare Workers During the COVID-19 Pandemic: A Systematic Review and Meta-Analysis. *Brain, Behavior, and Immunity*.

[2] Greenberg N., Weston D., Hall C., Caulfield T., Williamson V., Fong K. (2021). Mental Health of Staff Working in Intensive Care During COVID-19. *Occupational Medicine*.

[3] Rose S., Hartnett J., Pillai S. (2022). A Comprehensive Dataset Describing Nurses’ Emotions, Perceived Stressors and Coping Mechanisms During the First Surge of the COVID-19 Pandemic. *Data in Brief*.

[4] Abdulmohdi N. (2024). The Relationships Between Nurses’ Resilience, Burnout, Perceived Organisational Support and Social Support During the Second Wave of the COVID-19 Pandemic: A Quantitative Cross-Sectional Survey. *Nursing Open*.

[5] Manzano García G., Ayala Calvo J. C. (2021). The Threat of COVID-19 and Its Influence on Nursing Staff Burnout. *Journal of Advanced Nursing*.

[6] Kinman G., Teoh K., Harriss A. (2020). *The Mental Health and Wellbeing of Nurses and Midwives in the United Kingdom*.

[7] Van Camp J., Chappy S. (2017). The Effectiveness of Nurse Residency Programs on Retention: A Systematic Review. *AORN Journal*.

[8] Buchan J., Charlesworth A., Gershlick B. (2019). *A Critical Moment: NHS Staffing Trends, Retention and Attrition*.

[9] Royal College of Nursing (2023). *Valuing Nursing in the UK: Staffing for Safe and Effective Care in the UK: Interventions to Mitigate Risks to Nursing Retention*.

[10] West M., Bailey S., Williams E. *The Courage of Compassion Supporting Nurses and Midwives to Deliver high-quality Care*.

[11] Aiken L. H., Cerón C., Simonetti M. (2018). Hospital Nurse Staffing and Patient Outcomes. *Revista Médica Clínica Las Condes*.

[12] Griffiths P., Ball J., Bloor K. (2018). Nurse Staffing Levels, Missed Vital Signs and Mortality in Hospitals: Retrospective Longitudinal Observational Study. *Health Services and Delivery Research*.

[13] Rae P., Pearce S., Greaves P. J., Dall’Ora C., Griffiths P., Endacott R. (2021). Outcomes Sensitive to Critical Care Nurse Staffing Levels: A Systematic Review. *Intensive and Critical Care Nursing*.

[14] NHS England (2023). *NHS England Long Term Workforce Plan*.

[15] NHS England (2022). *NHS Health and Well-being Framework*.

[16] NHS England (2020). *People Promise*.

[17] World Health Organization (2021). *Comprehensive Mental Health Action Plan 2013–2030*.

[18] Lin S., Deng X., Ryan I. (2022). COVID-19 Symptoms and Deaths Among Healthcare Workers, United States. *Emerging Infectious Diseases*.

[19] World Health Organization (2021). *Health Systems Resilience During COVID-19: Lessons for Building Back Better*.

[20] NMC (2025). The NMC Register. *Nursing and Midwifery Council*.

[21] Universities and Colleges Admissions Service (UCAS) (2025). 2025 Cycle Applicant Figures–29 January Deadline: Available Online. https://www.ucas.com/data-and-analysis/undergraduate-statistics-and-reports/ucas-undergraduate-releases/applicant-releases-2025-cycle/2025-cycle-applicant-figures-29-january-deadline.

[22] RCM (2021). RCM Warns of Midwife Exodus as Maternity Staffing Crisis Grows. https://uat.rcm.org.uk/media-releases/2021/september/rcm-warns-of-midwife-exodus-as-maternity-staffing-crisis-grows/.

[23] NHS Digital (2021). NHS Workforce Statistics-April 2021 (Including Selected Provisional Statistics for May 2021). https://digital.nhs.uk/data-and-information/publications/statistical/nhs-workforce-statistics/april-2021.

[24] Dunkley-Bent J. (2017). A-EQUIP: The New Model of Midwifery Supervision. *British Journal of Midwifery*.

[25] NHS England (2021). *Professional Nurse Advocate. A-EQUIP Model: A Model of Clinical Supervision for Nurses*.

[26] Proctor B. (1986). Supervision: A co-Operative Exercise in Accountability. *Enabling and Ensuring: Supervision in Practice*.

[27] Douglas H., Ginty M. (2001). The Solihull Approach: Changes in Health Visiting Practice. *Community Practitioner*.

[28] Baird B., Murray R., Seale B., Foot C., Perry C. (2015). *Midwifery Regulation in the United Kingdom*.

[29] NHS England (2017). *A-EQUIP: A Model of Clinical Midwifery Supervision*.

[30] Rothwell C., Kehoe A., Farook S. F., Illing J. (2021). Enablers and Barriers to Effective Clinical Supervision in the Workplace: a Rapid Evidence Review. *BMJ Open*.

[31] Masamha R., Alfred L., Harris R., Bassett S., Burden S., Gilmore A. (2022). Barriers to Overcoming the Barriers: a Scoping Review Exploring 30 Years of Clinical Supervision Literature. *Journal of Advanced Nursing*.

[32] Engward H., Goldspink S., Abdulmodi N. (2024). Understanding Professional Advocacy: a Protocol for a Mixed Method Project to Explore Professional Nurse Advocacy and Professional Midwifery Advocacy in One NHS Trust. *SocArXiv*.

[33] Goldspink S., Engward E., Alexander M., Abdulmohdi N. (2024). Developing Sustainable Knowledge Partnerships: Joining the DOTS Between Inter-Organizational Research. *International Journal of Collaborative-Dialogic Practices*.

[34] Wallbank S., Hatton S. (2011). Reducing Burnout and Stress: The Effectiveness of Clinical Supervision. *Community Practitioner: The Journal of the Community Practitioners’ & Health Visitors’ Association*.

[35] Wallbank S. (2016). *The Restorative Resilience Model of Supervision A Reader Exploring Resilience to Workplace Stress in Health and Social Care Professionals*.

[36] The King’s Fund (2020). The Courage of Compassion: Supporting Nurses and Midwives to Deliver High-Quality Care. https://assets.kingsfund.org.uk/f/256914/x/ccc7dc0553/courage_of_compassion_summary_2020.pdf.

[37] Delgadillo J., Saxon D., Barkham M. (2018). Associations Between Therapists’ Occupational Burnout and Their Patients’ Depression and Anxiety Treatment Outcomes. *Depression and Anxiety*.

[38] Peterson U., Demerouti E., Bergström G., Samuelsson M., Åsberg M., Nygren Å. (2008). Burnout and Physical and Mental Health Among Swedish Healthcare Workers. *Journal of Advanced Nursing*.

[39] Laschinger H. K., Finegan J., Shamian J., Wilk P. (2001). Impact of Structural and Psychological Empowerment on Job Strain in Nursing Work Settings: Expanding Kanter’s Model. *The Journal of Nursing Administration*.

[40] Mansour M., Mattukoyya R. A. (2018). Cross-Sectional Survey of British Newly Graduated Nurses’ Experience of Organization Empowerment and of Challenging Unsafe Practices. *The Journal of Continuing Education in Nursing*.

[41] Kanter R. M. (2008). *Men and Women of the Corporation*.

[42] Zhang X., Ye H., Li Y. (2018). Correlates of Structural Empowerment, Psychological Empowerment and Emotional Exhaustion Among Registered Nurses: A Meta-Analysis. *Applied Nursing Research*.

[43] Fragkos K. C., Makrykosta P., Frangos C. C. (2020). Structural Empowerment is a Strong Predictor of Organizational Commitment in Nurses: A Systematic Review and meta-analysis. *Journal of Advanced Nursing*.

[44] Pettit A., Stephen R. (2015). *Supporting Health Visitors and Fostering Resilience: Literature Review*.

[45] Martin P., Lizarondo L., Kumar S., Snowdon D. (2021). Impact of Clinical Supervision on Healthcare Organisational Outcomes: A Mixed Methods Systematic Review. *PLoS One*.

[46] Ash G. L., Pollock J. (2023). Clinical Supervision Led by Professional Midwife Advocates. *Nursing*.

[47] McDonough J. H., Rhodes K., Procter N. (2024). Impact of Clinical Supervision on the Mental Health Nursing Workforce: A Scoping Review Protocol. *BMJ Open*.

[48] Armstrong S., Porter J. E., Larkins J. A., Mesagno C. (2022). Burnout, Stress and Resilience of an Australian Regional Hospital During COVID-19: A Longitudinal Study. *BMC Health Services Research*.

[49] Ahmed F., Bani-Issa W., Timmins F. (2022). Managing During the COVID-19 Pandemic: A Cross-Sectional Study of Health Care Workers’ Perceived Organizational Support and Its Consequences on Their Compassion, Resilience and Turnover Intention. *Journal of Nursing Management*.

[50] Lees-Deutsch L., Kneafsey R., Rodrigues A. (2023). National Evaluation of the Professional Nurse Advocate Programme in England: SUSTAIN: Supervision, Support, Advocacy for Improvement in Nursing, Mixed Methods Study.

[51] Smythe A., Flatt C., Mahachi L., Whatley V. (2023). Introduction of the Professional Nurse Advocate Role Using a Quality Implementation Framework. *British Journal of Nursing*.

[52] Wade R. (2023). Embedding the A-EQUIP Model of Restorative Supervision in a Critical Care Unit by Professional Nurse Advocates. *British Journal of Nursing*.

[53] Sharman V. L., Gadher A., Shipperlee F. (2024). Benefits and Challenges of Implementing the Professional Nurse Advocate Programme: A Service Evaluation. *Mental Health Practice*.

[54] Rouse S. (2019). The Role of the PMA and Barriers to the Successful Implementation of Restorative Clinical Supervision. *British Journal of Midwifery*.

[55] Baldwin S., Coyne T., Kelly P. (2022). Supporting Nursing, Midwifery and Allied Health Professional Teams Through Restorative Clinical Supervision. *British Journal of Nursing*.

[56] Davis F. D. (1989). Perceived Usefulness, Perceived Ease of Use, and User Acceptance of Information Technology. *MIS Quarterly*.

[57] Andersen R. (2008). National Health Surveys and the Behavioral Model of Health Services Use. *Medical Care*.

[58] Goldspink S., Tuckwell A., van Veggel N., Engward H., Abdulmohdi N., Alexander M. (2025). Professionals In-Place: The Role of the Practice-Based Research Coordinator. *Nurse Researcher*.

[59] Naing L., Nordin R. B., Abdul Rahman H., Naing Y. T. (2022). Sample Size Calculation for Prevalence Studies Using Scalex and Scalar Calculators. *BMC Medical Research Methodology*.

[60] Bakker A. B., Demerouti E. (2007). The Job Demands-Resources Model: State of the Art. *Journal of Managerial Psychology*.

[61] Varnhagen C. K., Gushta M., Daniels J. (2005). How Informed is Online Informed Consent?. *Ethics & Behavior*.

[62] Braun V., Clarke V. (2021). *Thematic Analysis: A Practical Guide*.

[63] Leone C., Bruyneel L., Anderson J. E. (2015). Work Environment Issues and Intention-to-Leave in Portuguese Nurses: A Cross-Sectional Study. *Health Policy*.

